# Bacterial glycobiology: rhamnose-containing cell wall polysaccharides in Gram-positive bacteria

**DOI:** 10.1093/femsre/fuw006

**Published:** 2016-03-13

**Authors:** Michel-Yves Mistou, Iain C. Sutcliffe, Nina M. van Sorge

**Affiliations:** 1 Laboratory for Food Safety, Université Paris-Est, ANSES, F-94701 Maisons-Alfort, France; 2 Department of Applied Sciences, Northumbria University, Newcastle upon Tyne, NE1 8ST, UK; 3 Medical Microbiology, University Medical Center Utrecht, 3584 CX Utrecht, The Netherlands

**Keywords:** cell wall polysaccharide, rhamnose, pathogenesis, biosynthesis, glycobiology, Gram-positive bacteria

## Abstract

The composition of the Gram-positive cell wall is typically described as containing peptidoglycan, proteins and essential secondary cell wall structures called teichoic acids, which comprise approximately half of the cell wall mass. The cell walls of many species within the genera *Streptococcus*, *Enterococcus* and *Lactococcus* contain large amounts of the sugar rhamnose, which is incorporated in cell wall-anchored polysaccharides (CWP) that possibly function as homologues of well-studied wall teichoic acids (WTA). The presence and chemical structure of many rhamnose-containing cell wall polysaccharides (RhaCWP) has sometimes been known for decades. In contrast to WTA, insight into the biosynthesis and functional role of RhaCWP has been lacking. Recent studies in human streptococcal and enterococcal pathogens have highlighted critical roles for these complex polysaccharides in bacterial cell wall architecture and pathogenesis. In this review, we provide an overview of the RhaCWP with regards to their biosynthesis, genetics and biological function in species most relevant to human health. We also briefly discuss how increased knowledge in this field can provide interesting leads for new therapeutic compounds and improve biotechnological applications.

## Abstract (precis)

This review summarizes new insights into the genetics and function of rhamnose-containing cell wall polysaccharides expressed by lactic acid bacteria, which includes medically important pathogens, and discusses perspectives on possible future therapeutic and biotechnological applications.

## INTRODUCTION

The composition of the bacterial cell wall is critical for fundamental features such as bacterial cell shape, protection from and interaction with the environment. Carbohydrates are the most abundant molecules in the Gram-positive cell wall with much of it incorporated in the thick layer of peptidoglycan (15–100 nm) that surrounds the cell membrane (Silhavy, Kahne and Walker 2010). Peptidoglycan is composed of alternating β-1,4-linked *N*-acetylglucosamine (GlcNAc) and *N*-acetylmuramic acid (MurNAc) polysaccharide strands that are cross-linked by short peptides to form a three-dimensional network. Individual species tailor important physical properties of their peptidoglycan such as elasticity and porosity through the composition of the peptide bridge, the amount of crosslinking and chemical modifications of the composing glycan residues (Vollmer, Blanot and de Pedro 2008; Vollmer and Seligman 2010). Peptidoglycan also acts as a scaffold for other critical cell wall structures. For example, proteins containing LPXTG amino acid motifs are covalently attached to the peptidoglycan peptide bridge through the enzymatic action of sortase A (Hendrickx *et al*. 2011).

Cell wall polysaccharides (CWP), such as capsular polysaccharides and wall teichoic acids (WTA), are anchored to peptidoglycan GlcNAc or MurNAc, covering the bacterium with a layer of glycans that is directly exposed to the environment (Deng *et al*. 2000; Swoboda *et al*. 2010; Yother 2011). The predominance of capsulated species among bacterial pathogens instigated studies on the role of capsular polysaccharides in infectious disease pathogenesis. As a result, effective capsule polysaccharide conjugate vaccines were developed against various species including *Neisseria meningitidis*, *Haemophilus influenzae* type B and *Streptococcus pneumoniae*. For some human bacterial pathogens, such as *Staphylococcus aureus* and *Streptococcus pyogenes*, the role of polysaccharide capsule in pathogenesis is less pronounced (O'Riordan and Lee 2004; Flores *et al*. 2012). In addition, the discovery of host pattern-recognition receptors with specificity for carbohydrates as well as technological advances in the area of complex carbohydrate analysis and synthesis have fueled initiatives to investigate bacterial glycobiology more comprehensively by unraveling the structure, biosynthesis and functions of bacterial polysaccharide structures.

In this review, we focus on a subclass of secondary CWP that we refer to as rhamnose-containing CWP (RhaCWP). L-rhamnose is commonly found in bacteria but is not used or produced by humans (Maki and Renkonen 2004; Adibekian *et al*. 2011). Interestingly, L-rhamnose is often essential for bacterial virulence or even viability (Maki and Renkonen 2004), making its biosynthesis pathway an attractive therapeutic target. We will therefore review the current knowledge regarding L-rhamnose biosynthesis and functions in bacteria in more detail. Recent insights into the genetic basis and function of RhaCWP in two important human pathogens, *S. pyogenes* (Group A *Streptococcus*) and *Streptococcus agalactiae* (Group B *Streptococcus*), have emphasized the critical role of these molecules in cell wall biogenesis and pathogenesis (Caliot *et al*. 2012; van Sorge *et al*. 2014). This functional information combined with the localization and abundance of RhaCWP suggests that parallels can be drawn with WTA in other Gram-positive bacteria. WTA structure and function have been reviewed extensively (Weidenmaier and Peschel 2008; Swoboda *et al*. 2010; Brown, Santa Maria and Walker 2013). Therefore, we will only highlight specific parallels with WTA biology throughout this review. Finally, from their historic discovery (Lancefield 1933) and recent insight from bacterial genome sequences, it is apparent that RhaCWP are likely more widespread in Gram-positive cocci within the order *Lactobacillales*. We will provide an overview of their inferred distribution and review the literature for selected species with regard to structure, genetics, biosynthesis and function. We will end by discussing the potential therapeutic and biotechnological applications of this important class of CWP.

### Non-classical CWP in Gram-positive cocci: a historical perspective

Most knowledge regarding the architecture and biology of the Gram-positive cell wall is derived from the model organisms *Bacillus subtilis* and *S. aureus*. For these and other species, WTA is a major cell wall component representing up to 60% of the total cell wall mass. WTA are anionic glycopolymers that are covalently attached to the peptidoglycan MurNAc residue (Swoboda *et al*. 2010). Most commonly, WTA are composed of a poly-ribitolphosphate (-RboP)- or poly-glycerolphosphate (-GroP-) backbone with modifications such as glycosylation and *D*-alanylation, the latter of which can neutralize the negative charge of the abundant phosphates in the WTA backbone (Brown, Santa Maria and Walker 2013). However, the exact chemical composition varies among and even within species (Neuhaus and Baddiley 2003; Weidenmaier and Peschel 2008; Winstel, Xia and Peschel 2014). It has long been recognized that not all Gram-positive bacteria incorporate polyRboP- or a polyGroP-based WTA into their cell wall. Instead, the cell walls of many species within the order *Lactobacillales* contain peptidoglycan-anchored polysaccharides that are characterized by the presence of L-rhamnose. Probably the first reports of RhaCWP in the genus *Streptococcus* date back to the late 1920s and early 1930s by Rebecca Lancefield (Lancefield 1928a,b, 1933). Her seminal work enabled the development of a new streptococcal classification system—in addition to hemolytic typing—based on the differential antigenic properties of streptococcal CWP, referred to as ‘C-substance’ or ‘Group Antigen’ (Lancefield 1933). The streptococcal serotyping scheme initially only discriminated Group A–E streptococci but was expanded to comprise as many as 20 serotypes called Groups A–V (excluding I and J) (Facklam 2002; Kohler 2007). The Lancefield typing system has been instrumental to link mild and severe human and animal diseases to specific bacterial groups within the genus *Streptococcus*, most notably *S. pyogenes*, causing more than 700 million infections resulting in over 500 000 deaths worldwide annually (Carapetis *et al*. 2005), and *S. agalactiae*, a pathogen affecting mainly neonates and the elderly (Phares *et al*. 2008; Edmond *et al*. 2012). Furthermore, the Lancefield typing scheme enabled the development of rapid diagnostic tests that aided clinically relevant ‘species identification’ (Lue, Howit and Ellner 1978). Increased recognition of additional strain characteristics, such as nutrient requirements and later the use of 16S rRNA for classification (and more recently whole genome sequencing), demonstrate that the Lancefield typing system cannot discriminate to the species level such that a single Lancefield Group represents one species. For example, the Group A antigen was long thought to be the exclusive molecular marker of *S. pyogenes*, yet *Streptococcus castoreus* is also noted to react with Group A antisera in commercial diagnostic kits (Lawson *et al*. 2005). Vice versa, a single species can express different Group antigens; strains of *Streptococcus dysgalactiae* subsp. *equisimilis* commonly express either the Group C or G antigen (Broyles *et al*. 2009; McMillan *et al*. 2010a; Takahashi, Ubukata and Watanabe 2011) and occasionally Group A carrying *S. dysgalactiae* subsp. *equisimilis* strains are identified (Tanaka *et al*. 2008; McMillan *et al*. 2010b). Consequently, the taxonomy and nomenclature of the genus *Streptococcus* has been reevaluated and reclassified over the years (Facklam 2002; Kohler 2007). This resulted in the split of the genus *Streptococcus* into three genera, i.e. *Enterococcus*, *Lactococcus* and *Streptococcus* (Schleifer *et al*. 1985), as well as subsequent description of several novel members within these genera. Two species covered in this review, *Enterococcus faecalis* and *Lactococcus lactis*, were formerly known as *Streptococcus faecalis* (Lancefield Group D) and *Streptococcus lactis* (Lancefield Group N). Today, more than 150 different species are known within these three genera, which remain classified within the order *Lactobacillales* (http://www.bacterio.net/-classifphyla.html#Firmicutes; Price *et al*. 2012).

Although the direct correlation between Lancefield serotyping and species clearly no longer persists, bioinformatic analyses of genome sequences indicates that many of the species within these three genera express RhaCWP (see section ‘*Distribution of RhaCWP throughout bacteria’*). We focus here on the species relevant to food production and human health and with experimental evidence for the presence of RhaCWP: Lancefield Groups A, B, C, E and G *Streptococcus* represented by *S. pyogenes*, *S. agalactiae*, *Streptococcus equi* subsp. *zooepidemicus*, *Streptococcus mutans* and *S. dysgalactiae* subsp. *equisimilis*, respectively, as well as *E. faecalis* and *L. lactis*.

### Cell wall organization of RhaCWP

**Figure 1.**
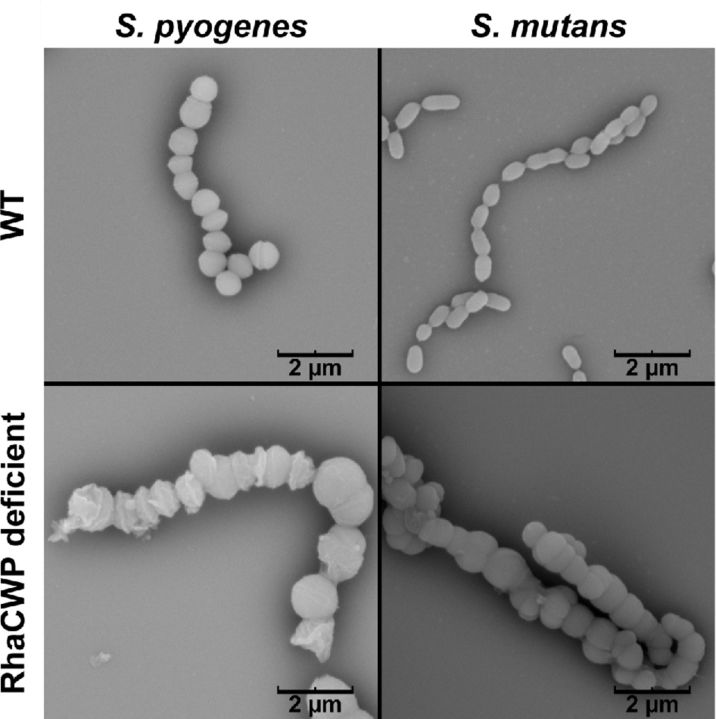
Cell division and separation defects caused by RhaCWP deficiency in *S. mutans* and *S. pyogenes*. Representative scanning electron microscopy images of *S. pyogenes* and *S. mutans* wild-type (WT) strains and corresponding RhaCWP-deficient strains. In *S. pyogenes* loss of the GAC was enforced by inducible knockout of *gacA* (*rmlD* homologue) and in *S. mutans*, deletion of *rmlD* results in loss of RhaCWP (van der Beek *et al*. 2015). Scale bar is indicated in image.

The RhaCWP is a major component in streptococcal species comprising about 40%–60% of the cell wall by weight (McCarty 1952; Krause and McCarty 1962a; Krause 1963; Doran and Mattingly 1982). Correspondingly, cell wall thickness is visually reduced by 40%–50% after chemical extraction of the Group-specific carbohydrate (Swanson and Gotschlich 1973; Wagner and Wagner 1978). Recent mutagenesis studies confirmed the structural importance of streptococcal group antigens; complete loss of RhaCWP expression resulted in severe growth and cell division abnormalities (Fig. 1; Tsukioka *et al*. 1997a; Caliot *et al*. 2012; van Sorge *et al*. 2014; van der Beek *et al*. 2015).

**Figure 2.**
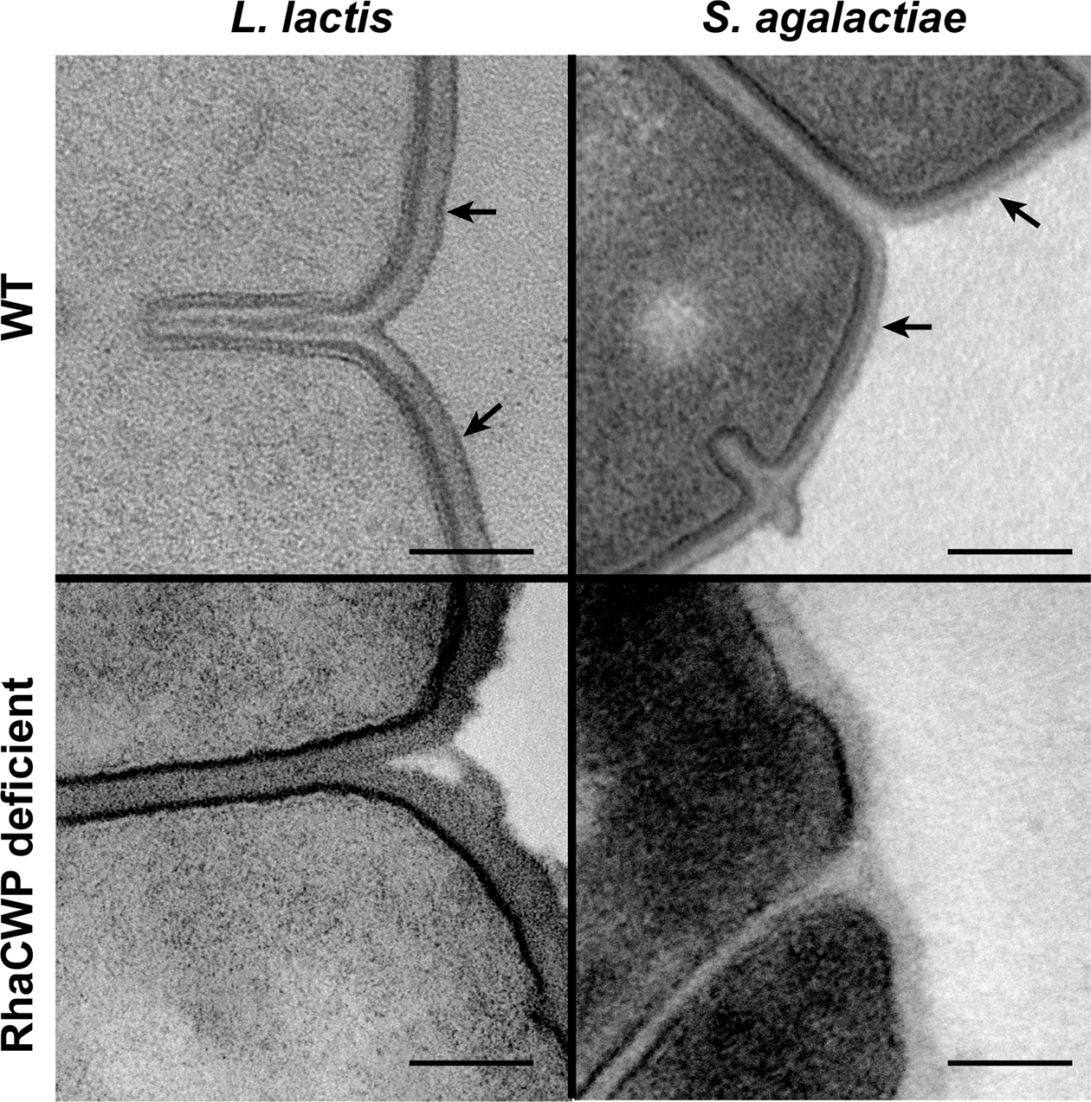
Visualization of the pellicle by transmission electron microscopy. *Lactococcus lactis* and *S. agalactiae* transmission electron microscope images contrasted with heavy metal staining method. In wild-type (WT) strains the pellicle is visible and indicated by arrows. Loss of RhaCWP expression, due to of genetic mutation, result in loss of pellicle expression. The scale bar represents 0.1 μm.

RhaCWP are localized on the outermost surface of the cell wall but are likely also intercalated within the mesh-like structure of the peptidoglycan layer since antibodies directed against these structures bind to both sides of isolated cell walls (Swanson and Gotschlich 1973; Wagner and Wagner 1978; Wagner *et al*. 1980). Group-specific antigens can also be isolated at high yield in the growth medium (Carey *et al*. 1980; De Cueninck, Shockman and Swenson 1982; Doran and Mattingly 1982), possibly as a result of cell wall catabolism during growth. Compared to the cell walls of *S. aureus* and *B. subtilis*, streptococcal species appear to lack expression of polyol-based WTA and, typically, lack orthologues of the critical WTA biosynthesis enzymes TagB, TagD and TagF (Sutcliffe, Black and Harrington 2008) (and unpublished observations). In contrast to streptococci, homologues of WTA biosynthesis genes are found in lactococcal genomes. Consequently, *L. lactis* strains likely express both polyol-based WTA and RhaCWP as part of their cell wall (Chapot-Chartier and Kulakauskas 2014). *E. faecalis* expresses the most extensive surface glycome including WTA, LTA, capsular polysaccharide and a RhaCWP called Enterococcal polysaccharide antigen (Epa) (Hancock and Gilmore 2002; Teng *et al*. 2009; Thurlow, Thomas and Hancock 2009; Theilacker *et al*. 2012). In contrast to RhaCWP in streptococci and *L. lactis*, Epa appears to be buried in the cell wall precluding interaction with the immune system, at least under laboratory conditions (Hancock and Gilmore 2002). It should however be noted that sera from patients do contain Epa-specific antibodies (Xu, Murray and Weinstock 1998; Xu *et al*. 2000; Teng *et al*. 2002). Also, the presence of Epa is visible under transmission electron microscopy (TEM) as a separate electron dense outer layer (Rigottier-Gois *et al*. 2015), similar to the RhaCWP layer observed in *L. lactis* and *S. agalactiae* (Chapot-Chartier *et al*. 2010; Caliot *et al*. 2012) (Fig. 2). In early studies, this electron dense outer layer was known as the outer lamina and was initially described as microcapsule (Baker and Kasper 1976). Recently, the term pellicle was coined (Chapot-Chartier *et al*. 2010) and is preferred since the term does not inherently imply that this layer is either ‘outer’ or of a specific composition (such as capsule). Interestingly, the pellicle is not as impenetrable as TEM images suggest. In *S. agalactiae*, topographic imaging and atomic force microscopy-based single-molecule mapping on live bacteria revealed that peptidoglycan strands can still be probed in the presence of the GBC (Beaussart *et al*. 2014). However, the pellicle does shield peptidoglycan to some extent, since loss of GBC expression through genetic manipulation renders *S. agalactiae* extremely sensitive to the activity of peptidoglycan-cleaving mutanolysin (Caliot *et al*. 2012). Similarly, atomic force microscopy studies on pellicle-deficient *L. lactis* uncovers peptidoglycan periodic bands orientated parallel to the short axis of the cell (Andre *et al*. 2010).

### Chemical structure of RhaCWP

**Figure 3.**
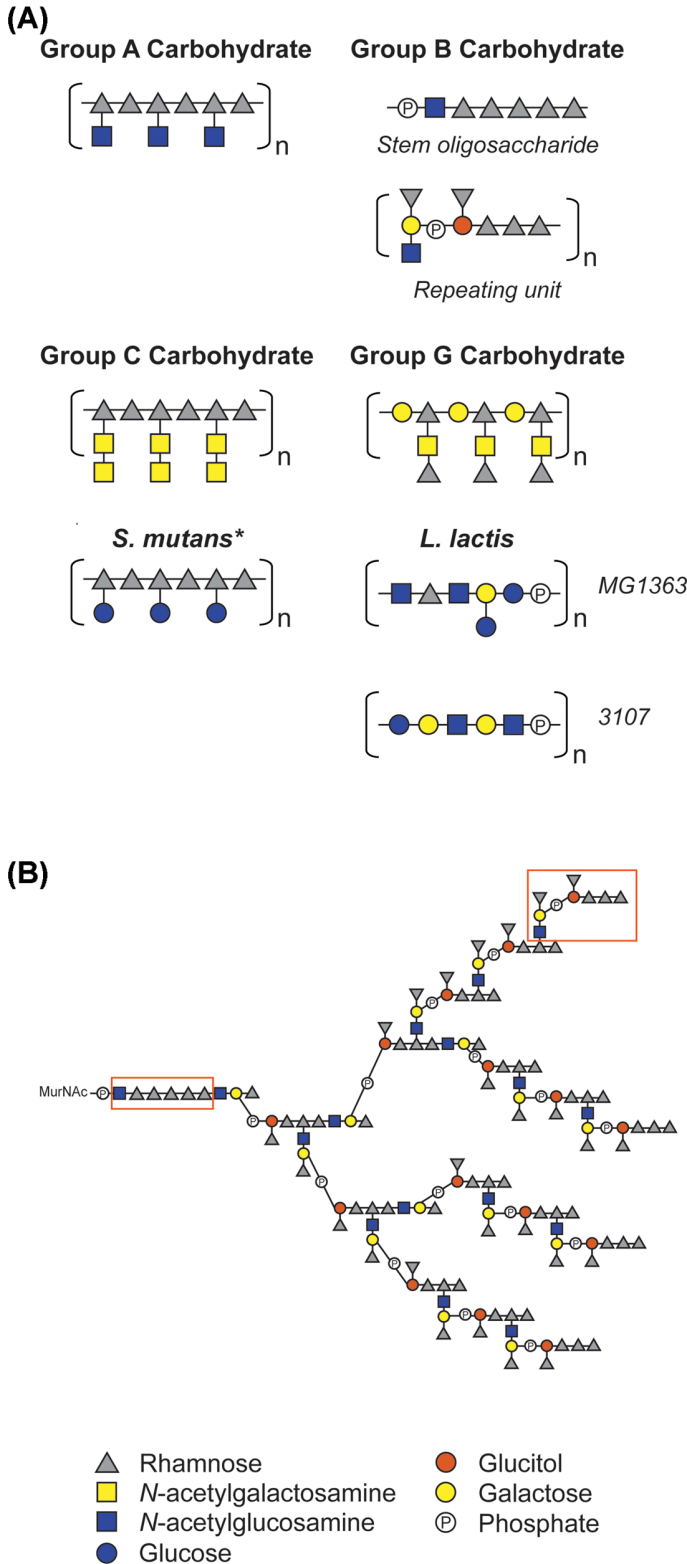
Schematic representation of known RhaCWP structures. (**A)** Schematic representation of RhaCWP core structures in different streptococcal species and *L. lactis*. GAC structure (McCarty and Lancefield 1955; Coligan, Kindt and Krause 1978; Pritchard *et al*. 1982; Huang, Rama Krishna and Pritchard 1986); GBC structure (Pritchard, Gray and Dillon 1984; Michon *et al*. 1987; 1988); GCC structure (Krause and McCarty 1962a; Coligan, Kindt and Krause 1978); GGC structure (Pritchard *et al*. 1988); *S. mutans* serotypes c, e, f, k (Pritchard and Furner 1985; Pritchard *et al*. 1986, 1987; Nakano and Ooshima 2009); *L lactis* (Chapot-Chartier *et al*. 2010; Ainsworth *et al*. 2014; Chapot-Chartier and Kulakauskas 2014). *For *S. mutans* the glucose side chain can either be absent (serotype k) or be linked to the rhamnan backbone in α-1,2 configuration (serotype c), β-1,2 configuration (serotype e), or α-1,3 configuration (serotype f). It must be noted that the (distribution of) length(s) of the RhaCWP have not been experimentally determined. RhaCWP are likely covalently attached to peptidoglycan MurNAc. **(B)** Full structure of GBC as described by (Pritchard, Gray and Dillon 1984; Michon *et al*. 1987, 1988) with newly recognized structural elements highlighted in boxes. Because both the repeating unit and the rhamnan stem have a basal GlcNAc moiety, we hypothesize that the synthesis of each building block is initiated separately on the undecaprenyl lipid carrier by GbcO. It is recognized that either incomplete substitution (at branch points located on the penultimate rhamnose of the repeating unit) or further extension could create a more heterogeneous final structure than presented here. Phosphate groups are involved in phosphodiester bonds linking oligosaccharides into polysaccharides.

Studies focusing on the chemical composition of Lancefield Group antigens have demonstrated that rhamnose is the major constituent, along with variable combinations and linkages of Glc, GlcNAc, Gal, GalNAc and phosphate (Pritchard *et al*. 1981). Elucidation of Group-specific RhaCWP structures in individual streptococcal species provided structural evidence for the discriminating capacity of the Lancefield typing scheme (Fig. 3A). The serological distinction between Group A and Group C *Streptococcus* is explained by expression of a terminal β-linked GlcNAc side chain in the Group A Carbohydrate (GAC) versus a (GalNAc)_2_ side chain in the Group C Carbohydrate (GCC), respectively (McCarty 1956; Krause and McCarty 1962a; Coligan, Kindt and Krause 1978) (Fig. 3A). However, biochemical and immunological characterization of CWP isolated from so-called Group A- and Group C-variant strains also noted that the GAC and GCC are structurally related. These variant strains lost Lancefield serum reactivity and displayed a variant Group antigen comprised of unsubstituted rhamnan with only trace amount of the *N*-acetylated sugars (McCarty 1956; Krause and McCarty 1962b). Occurrence of such variant strains appears to be a rare event; one Group C-variant strain was isolated as a resistant clone after exposure to virulent Group C bacteriophages (Krause 1963), whereas Group A-variant strains appear after multiple passages through an unnatural host, such as mice, but have never been isolated from humans (McCarty and Lancefield 1955). Whether strains are able to vary expression, composition or length of RhaCWP during natural infection is currently unclear. Possibly such variants may be missed in routine diagnostics screening. Alternatively, loss of specific RhaCWP epitopes or loss of the complete structure would severely hamper the ability of the bacterium to colonize or infect the host, allowing rapid eradication by the host immune system as will be described in more detail below.

For the Group B (GBC) and G carbohydrate (GGC), rhamnose is the major antigenic determinant (Curtis and Krause 1964a,b). Species carrying these structures are serologically discriminated based on the presence of either a single rhamnose in GGC versus triterminal rhamnose in GBC (Curtis and Krause 1964a) (Fig. 3A and B). Among the streptococcal group antigens, the GBC is unique since it forms a multiantenna branching structure and is negatively charged, due to the presence of phosphodiester bonds that link different GBC repeat units (Fig. 3B). Similarly, the *L. lactis* RhaCWP contains phosphodiester bonds that link hexasaccharide or pentasaccharide repeating units (Fig. 3A) (Chapot-Chartier *et al*. 2010; Ainsworth *et al*. 2014). Unfortunately, the structure of *E. faecalis* Epa has not yet been elucidated but is composed of the monosaccharides glucose, rhamnose, GlcNAc, GalNAc, galactose and probably phosphate (Hancock and Gilmore 2002; Teng *et al*. 2009). The presence of phosphates in RhaCWP of *S. agalactiae*, *L. lactis* and possibly *E. faecalis* likely suggests that the functions of these structures more closely resemble functions exerted by WTA in other Gram-positive bacteria (Michon *et al*. 1987, 1988, 1991; Sutcliffe 2008).

### General aspects of RhaCWP genetics and biosynthesis

CWP can be structurally highly complex due the number and variation in their monosaccharide components, diverse linkage types and chemical substitutions such as (de)acetylation and hydroxylation. Their biosynthesis requires the coordinated action of glycosyltransferases (enzymes that link monosaccharides), transporters and metabolic enzymes for the production of nucleotide-sugar precursors. Often, genes encoding proteins required for glycoconjugate biosynthesis are clustered on the chromosome. Their identification allows subsequent structure–function studies that help to understand the role of glycosylation in bacterial (infection) biology.

It is surprising that despite the historical and medical importance of the Lancefield Group antigens, no studies were undertaken to decipher the genetic basis of their biosynthesis. By contrast, capsule synthesis was quickly recognized as a major virulence factor in many bacterial species and has consequently been the subject of many genetic, biochemical and functional studies (Llull, Lopez and Garcia 2001; Cress *et al*. 2014). Initial predictions for the genetic basis of Lancefield Group carbohydrate biosynthesis were postulated upon availability of the first streptococcal genome sequences (Ferretti *et al*. 2001; Glaser *et al*. 2002; Holden *et al*. 2009; Shimomura *et al*. 2011). For *S. agalactiae*, the availability of the GBC structure and genome sequence enabled a comprehensive and detailed *in silico* analysis that linked protein-encoding genes to the different glycosidic linkages in the GBC structure (Sutcliffe, Black and Harrington 2008). That study demonstrated that most, but not all, of the enzymatic activities for synthesis and transport of the mature GBC molecule are present in the predicted 15 kB GBC gene cluster (Sutcliffe, Black and Harrington 2008); proteins required for lipid carrier activation and peptidoglycan anchoring of GBC seemed to be encoded elsewhere on the genome (Sutcliffe, Black and Harrington 2008). As will be discussed below, this split in gene organization, correlating with different biosynthetic steps, appears to be a common feature of the streptococcal and lactoccocal RhaCWP biosynthesis pathway. In addition, the biosynthesis of RhaCWP requires the production of appropriate nucleotide sugars precursors. We will only cover dTDP-L-rhamnose biosynthesis here given its characteristic presence in RhaCWP, as well as the possible therapeutic implications of this pathway. We will then discuss the genetic organization and putative biosynthesis pathway of RhaCWP.

### L-rhamnose biosynthesis

**Figure 4.**
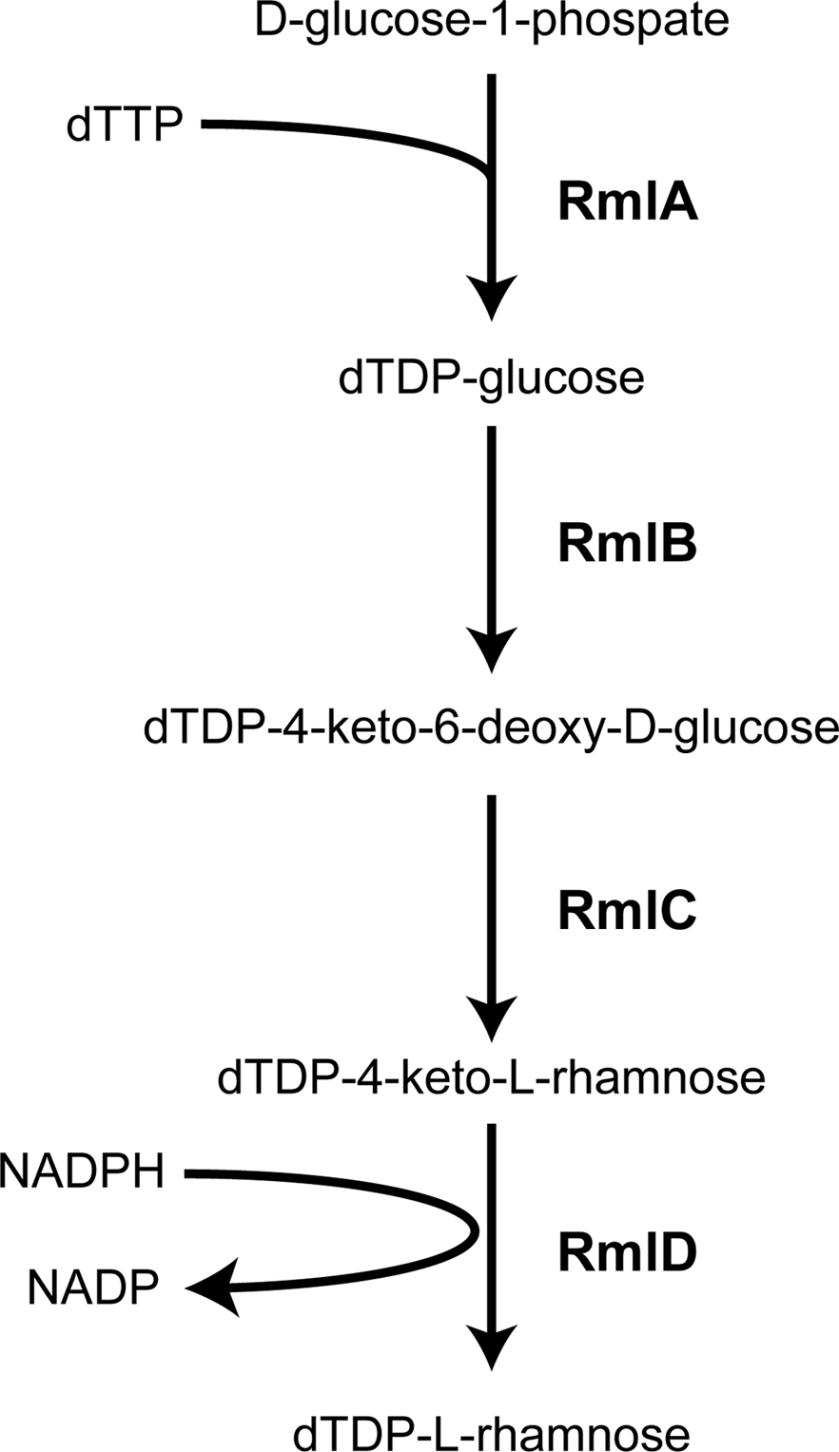
L-rhamnose biosynthesis pathway. Four-step catalytic reaction resulting in dTDP-L-rhamnose production from D-glucose-1-phosphate.

Incorporation of L-rhamnose into polysaccharide structures in both Gram-positive and Gram-negative bacteria requires the formation of the nucleotide sugar precursor dTDP-L-rhamnose. dTDP-L-rhamnose is produced from glucose-1-phophate through a conserved four-step enzymatic reaction that has been characterized both biochemically and structurally (Fig. 4) (Giraud and Naismith 2000; Dong *et al*. 2003a). In the first step of the pathway, RmlA, a glucose-1-phosphate thymidyltransferase, converts glucose-1-phosphate into dTDP-glucose (Blankenfeldt *et al*. 2000), which is subsequently oxidized and dehydrated to form dTDP-4-keto-6-deoxy-D-glucose by the dTDP-D-glucose 4,6-dehydratase RmlB (Beis *et al*. 2003). RmlC catalyzes an unusual double epimerization reaction (Giraud *et al*. 2000; Dong *et al*. 2003b; 2007), the product of which is finally reduced by RmlD, a dTDP-4-dehydrorhamnosereductase, to form dTDP-L-rhamnose (Blankenfeldt *et al*. 2002; van der Beek *et al*. 2015). Initial structure elucidation of the Rml enzymes from different species, including *Pseudomonas aeruginosa* (RmlA), *Salmonella enterica* (RmlC and RmlD) and *Streptococcus suis* (RmlB and RmlC), demonstrated that rhamnose biosynthesis enzymes require dimerization or even dimerization of dimers to catalyze the respective enzymatic reactions (Blankenfeldt *et al*. 2000; Giraud *et al*. 2000; Blankenfeldt *et al*. 2002; Beis *et al*. 2003; Dong *et al*. 2007). However, recent structural and biochemical characterization of the *S. pyogenes* RmlD homologue provided surprising insight that, in this species, RmlD is active as a monomer (van der Beek *et al*. 2015). Subsequent comprehensive bioinformatics analysis of 213 putative RmlD sequences indicates that the monomeric form of RmlD is more widespread throughout the bacterial kingdom compared to the originally described RmlD from *Salmonella* (Blankenfeldt *et al*. 2002; van der Beek *et al*. 2015). The benefit of either structure to the enzymatic reaction is currently unknown.

In many bacteria, such as in *E. faecalis* (Xu, Murray and Weinstock 1998), *Shigella flexneri* (Macpherson, Manning and Morona 1994), *L. lactis* (Dupont *et al*. 2004), *S. pneumoniae* (Bentley *et al*. 2006) and *S. enterica* (Jiang *et al*. 1991), the RmlA-D proteins are encoded by a single genetic locus, *rmlABCD*. The *rml* genes are likely transcribed as an operon, although experimental evidence for that is currently lacking. In contrast, in some bacteria the *rml* genes display a split architecture, often clustering *rmlABC*, but excluding *rmlD*. This is for example the case in *Mycobacterium tuberculosis* (Ma *et al*. 2001) and several streptococcal species (Tsukioka *et al*. 1997a,b; van der Beek *et al*. 2015). The evolutionary origin or functional benefit of a split versus clustered gene architecture is remains to be determined.

The contribution of Rml enzymes to L-rhamnose biosynthesis has been demonstrated in several species through a genetics approach, i.e. mutation of any of the *rml* genes results in loss of L-rhamnose in the bacterial cell walls (Tsukioka *et al*. 1997a,b; Rahim *et al*. 2000; Carvalho *et al*. 2015; van der Beek *et al*. 2015). For other species, loss of L-rhamnose in the cell wall was not confirmed by cell wall composition analysis upon mutation of *rml* genes (Chiang and Mekalanos 1999; Xu *et al*. 2000). Importantly, disruption of dTDP-L-rhamnose biosynthesis severely attenuates bacterial fitness and/or virulence (Tsukioka *et al*. 1997a; Chiang and Mekalanos 1999; Rahim *et al*. 2000; Xu *et al*. 2000; Carvalho *et al*. 2015; van der Beek *et al*. 2015). The position or percentage of incorporated L-rhamnose likely dictates whether *rml* genes are essential. For example, in *M. tuberculosis*, L-rhamnose covalently links arabinogalactan to peptidoglycan, which is critical for the overall architecture of the Mycobacterial cell wall, making L-rhamnose biosynthesis essential (McNeil, Daffe and Brennan 1990; Ma, Pan and McNeil 2002). Similarly in *S. pyogenes*, L-rhamnose is incorporated in the GAC that comprises half of the cell wall mass (McCarty 1952), rendering depletion lethal (Le Breton *et al*. 2015; van der Beek *et al*. 2015). In the case of uropathogenic *Escherichia coli* and *P. aeruginosa*, mutation of *rmlD* results in loss of O-antigen expression but leaves the lipopolysaccharide core and lipid A structure intact, yielding viable bacteria (Burns and Hull 1998; Rahim *et al*. 2000). However, loss of rhamnose does come at a certain cost, since rhamnose-deficient *E. coli* are extremely sensitized to serum-mediated killing (Burns and Hull 1998).

**Figure 5.**
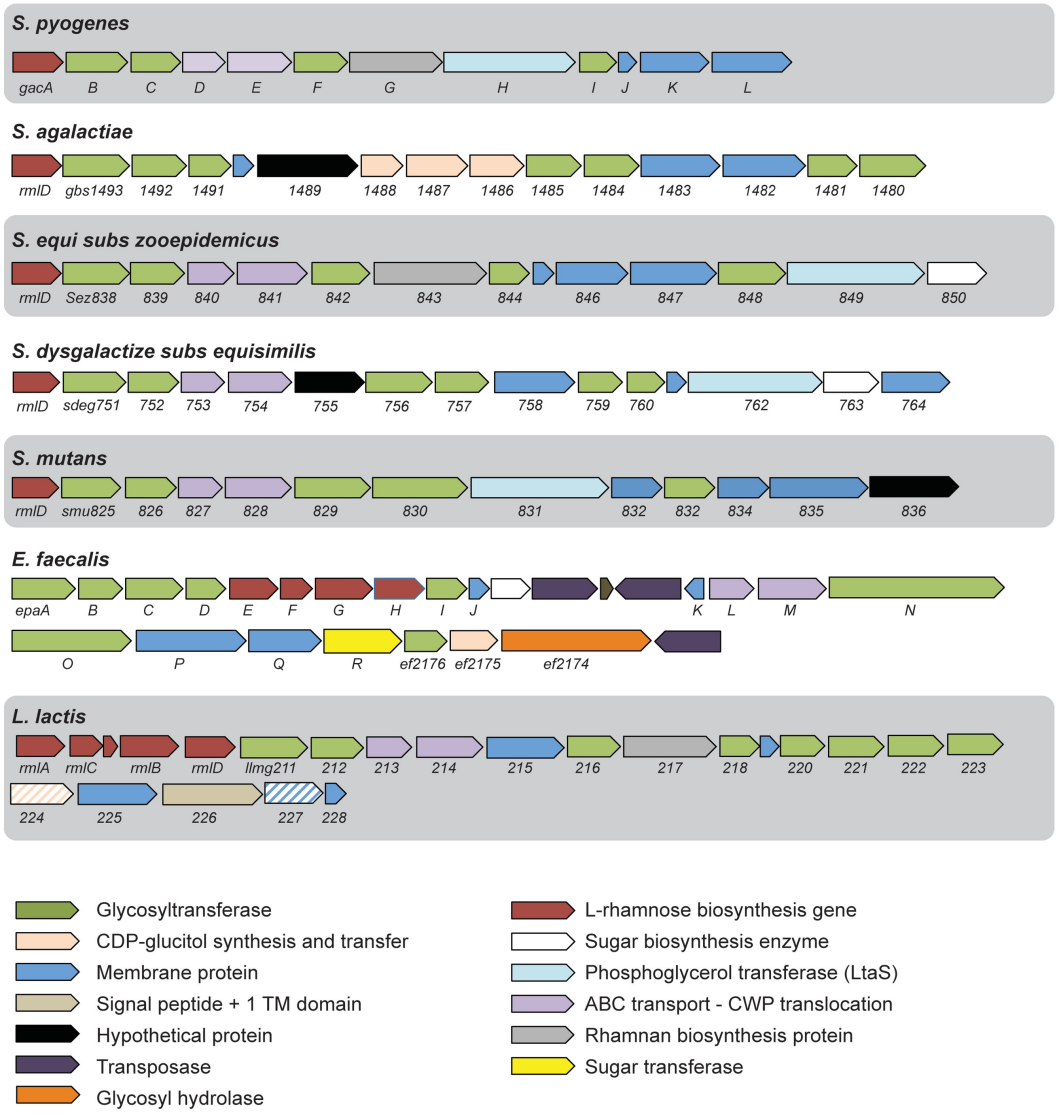
Comparison of the RhaCWP biosynthesis gene clusters in streptococcal species, *L. lactis* and *E. faecalis*. The RhaCWP biosynthesis genes are often located in *rmlD*-associated gene clusters. The loci vary between 14 and 26 kB in size encoding between 12 and 25 genes with annotated functions such as glycosyltransferases, polysaccharide biosynthesis proteins, rhamnose biosynthesis proteins (Rml proteins) and putative transport molecules. Each function is indicated by a different color. A representative gene cluster of a single species is presented and abbreviated gene annotations are indicated below. Arrows are drawn to scale with gene size. The RhaCWP gene clusters of the following strains are shown: *S. pyogenes* strain M5005 *spy0602 – spy0613*; *S. agalactiae* NEM316 *gbs1480 – gbs1494*; *S. equi subs equisimilis* MGCS10565 *Sez837 – Sez850*; *S. dysgalactiae sub equisimilis* GGS_124 *SDEG750 – SDEG764*; *S. mutans* UA159 *SMU.824 – SMU.836*; *E. faecalis* V583 *EF2198 – EF2174*; *L. lactis* MG1363 *llmg0206 – llmg0228*. TM, transmembrane

Analysis of its genomic location in members of the family *Streptococcaceae* and in *E. faecalis* reveal that *rmlD* is systematically associated with a large cluster of genes encoding glycosyltransferases, polysaccharide transport systems, sugar biosynthesis enzymes and genes of unknown functions (Fig. 5). Experimental evidence in selected streptococcal species, *L. lactis* and *E. faecalis* confirms that *rmlD*-associated loci participate in the biosynthesis of RhaCWP as will be discussed in the next section.

### Biosynthesis of RhaCWP

**Figure 6.**
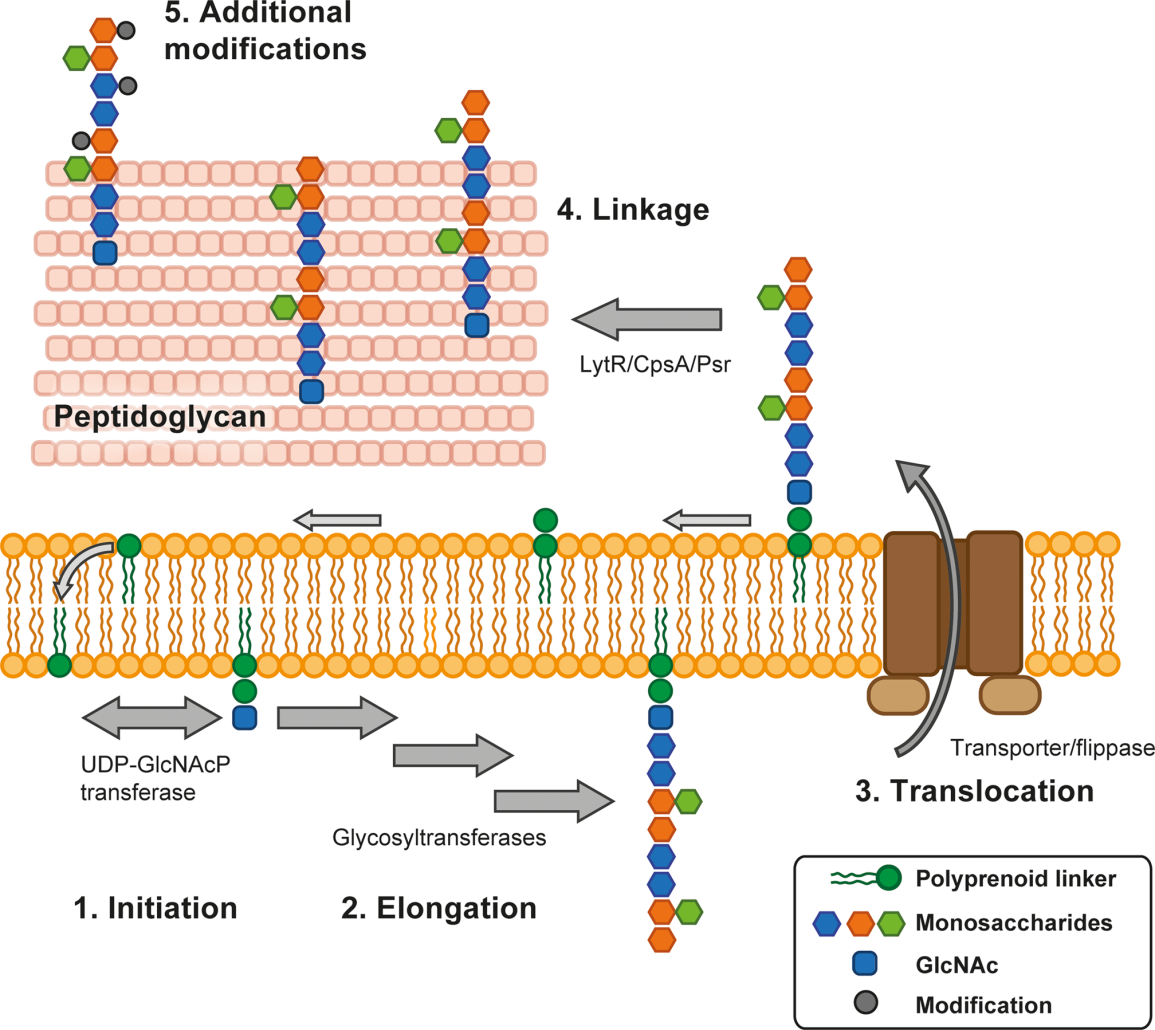
General steps in glycoconjugate biosynthesis. Despite glycoconjugate diversity, bacterial glycoconjugate biosynthesis is quite conserved and proceeds in five basic steps: (1) **initiation** of biosynthesis through activation of a lipid carrier on the cytoplasmic side of the membrane, (2) **elongation** by sequential addition of activated monosaccharides by glycosyltransferases to form the polysaccharide on the polyprenoid linker, (3) **translocation** of lipid-linked precursors across the membrane by ABC transporters or ‘flippases’, (4) **linkage** of the glycoconjugate to peptidoglycan by LytR/CpsA/Psr proteins, and (5) **additional modifications** to the glycoconjugate that can occur after anchoring to the cell wall, for example alanylation of wall teichoic acid. GlcNAc, *N*-acetylglucosamine. UDP-GlcNAcP transferase, UDP-GlcNAc:lipid phosphate transferase

Biosynthesis of glycosylated surface structures in bacteria displays some common themes, despite the considerable diversity in the chemical composition of glycosylated structures (reviewed in Tytgat and Lebeer 2014). In general, glycoconjugate biosynthesis proceeds as follows (Fig. 6); (1) **initiation** of biosynthesis through activation of a lipid carrier, often undecaprenylphosphate, on the cytoplasmic side of the membrane, (2) **elongation** of the polysaccharide (building block) on the lipid carrier by sequential addition of activated sugar precursors, (3) **translocation** of lipid-linked precursors, either repeating units or the complete glycoconjugate, across the membrane by ABC transporters or ‘flippases’ (Lazarevic and Karamata 1995), (4) **linkage** of the glycoconjugate to peptidoglycan or protein (Kawai *et al*. 2011; Chan *et al*. 2013) and (5) **additional modifications** to the glycoconjugate that can occur after cell wall anchoring. In some cases, the mature glycoconjugate is formed by polymerization of translocated repeating units between steps (3) and (4) by a dedicated polymerase. Since undecaprenylphosphate serves as a common scaffold to build structurally diverse glycoconjugates including capsules, peptidoglycan, lipopolysaccharides and protein-modifying glycans, the availability of ‘free’ undecaprenylphosphate is essential for bacterial survival (Hartley and Imperiali 2012).

In accordance with the general steps of glycoconjugate biosynthesis (Fig. 6), biosynthesis of RhaCWP is likely initiated on the inside of the cytoplasmic membrane on an undecaprenylphosphate lipid carrier, before the structure is transported across the membrane and attached to peptidoglycan. Indeed, most enzymes involved in RhaCWP biosynthesis are predicted to be intracellular or incorporated in the membrane. Furthermore, lysed protoplasts of *S. pyogenes* incorporate ^14^C-labeled dTDP-rhamnose into a polysaccharide structure, presumably the GAC (Zeleznick *et al*. 1963). The proteins required for elongation and translocation of RhaCWP appear to be encoded by *rmlD*-associated gene clusters (Fig. 5). Within each cluster, we find genes encoding glycosyltransferases, putative transport systems (either an ABC transport system or putative flippase), sugar modifying enzymes for the production of specific sugar precursors, and hypothetical proteins which can be unique for the species (Sutcliffe, Black and Harrington 2008). Enzymes for initiation (Fig. 6; step 1) and peptidoglycan anchoring after transport (Fig. 6; step 4) are encoded elsewhere on the genome in most species. It is beyond the scope of this review to discuss the putative role of each gene in detail; instead, we aim to provide genetic and biochemical insights concerning the proposed steps of the RhaCWP biosynthesis pathway.

#### Lipid carrier activation by UDP-GlcNAc:lipid phosphate transferases

The available structures of RhaCWP as well as evidence for their covalent linkage to peptidoglycan MurNAc (Heymann, Manniello and Barkulis 1967; Deng *et al*. 2000) implies that activation of the lipid carrier undecaprenylphosphate by the transfer of GlcNAc by a UDP-GlcNAc:lipid phosphate transferase is the first enzymatic step of RhaCWP biosynthesis. This step is similar to the initiating reaction for WTA biosynthesis in *B. subtilis* and *S. aureus*, which is catalyzed by integral membrane proteins called TarO/TagO (Swoboda *et al*. 2010). A gene encoding a TarO/TagO homologue is not present in the *rmlD*-associated gene clusters in streptococci and *L. lactis* (Fig. 5), but can be readily identified elsewhere in the genome through homology searches. Atypically in *E. faecalis*, the first gene of the putative RhaCWP gene cluster, *epaA*, may encode the required UDP-GlcNAc:lipid phosphate transferase (Fig. 5). Experimental proof for a role of these enzymes in the biosynthesis of RhaCWP has been provided for *S. pyogenes GacO*, *S. agalactiae GbcO*, *S. mutans RgpG* and *E. faecalis EpaA* through genetic mutation, bacterial complementation studies or pharmacological inhibition of the transferase by the compound tunicamycin (Yamashita *et al*. 1999; Teng *et al*. 2009; Campbell *et al*. 2011; Caliot *et al*. 2012; van Sorge *et al*. 2014). Indeed, interference with expression or enzymatic function of these transferases depleted the cell wall of L-rhamnose, attenuated bacterial growth and induced aberrant morphology and cell division resulting in an increased chain length (Yamashita *et al*. 1999; Teng *et al*. 2009; Caliot *et al*. 2012; van Sorge *et al*. 2014). Correspondingly, the *S. agalactiae gbcO* mutant lost expression of the pellicle (Fig. 2) (Caliot *et al*. 2012), confirming previous observations in *L. lactis* that this outer layer correlates with the presence of RhaCWP (Fig. 2) (Chapot-Chartier *et al*. 2010). For streptococci, this phenotype mimics the defects observed in *rml* mutants, which also lose expression of RhaCWP and display attenuated growth, aberrant morphology and growth in long chains (Fig. 1) (Tsukioka *et al*. 1997a; van der Beek *et al*. 2015).

#### Glycosyltransferases required for RhaCWP biosynthesis

Following lipid carrier activation by UDP-GlcNAc:lipid phosphate transferases, biosynthesis of the actual RhaCWP structure occurs through the step-wise addition of monosaccharides by specific glycosyltransferases. In most cases, each glycosidic bond requires a dedicated enzyme. Such knowledge informs detailed structure–function studies of RhaCWP as will be discussed below.

The Lancefield GAC, GCC and *S. mutans* RhaCWP contain a backbone composed of α-1,2-/α-1,3-linked polyrhamnose (Fig. 3A). The first seven genes of the respective RhaCWP gene clusters contain a high sequence identity, suggesting that these genes are required to construct the identical rhamnan backbone (Fig. 5). Indeed, heterologous expression of these seven genes of *S. mutans* in *E. coli* results in the production of α-1,2-/α-1,3-linked polyrhamnose (Shibata *et al*. 2002). Subsequently, Shibata *et al*. identified that three glycosyltransferases encoded in this partial cluster, *RgpA*, *RgpB* and *RgpF*, are required for rhamnan biosynthesis, with RgpA adding the first rhamnose to the undecaprenylphosphate-GlcNAc lipid carrier (Shibata *et al*. 2002). Homologous glycosyltransferases in *S. pyogenes* and *S. zooepidemicus* likely catalyze a similar reaction. Decoration of the rhamnan backbone with specific side chains produces the discriminating epitopes of the GAC, GCC and RhaCWP of *S. mutans* (Fig. 3A). The remaining glycosyltransferases in the respective gene clusters are likely implicated in these reactions. For *S. mutans*, RgpE and RgpI add α1,2-linked glucose to RhaCWP (Yamashita *et al*. 1998; Ozaki *et al*. 2002), whereas GacI in *S. pyogenes* likely adds the characteristic β-linked GlcNAc side chain since mutation of *gacI* results in loss of side chain expression (van Sorge *et al*. 2014). For construction of the GCC side chain, a disaccharide GalNAc (Fig. 3A), glycosyltransferases encoded by *Sez_0844* and *Sez_0848* likely play a crucial role. Similar to the GAC, GCC and RhaCWP of *S. mutans*, the Lancefield GGC is also reported to be a linear structure (Pritchard *et al*. 1988), albeit with a different disaccharide backbone (Fig. 3A). No studies have experimentally addressed the contribution of specific glycosyltransferases to the biosynthesis of the GGC, but the increased number of glycosidic bonds compared to the GAC, GCC and *S. mutans* RhaCWP, suggest that the activity of all seven glycosyltransferases encoded in the gene cluster (Fig. 5) are required for its biosynthesis.

The situation is more complex in the case of the pellicle-forming GBC and RhaCWP of *L. lactis* strains. The GBC was previously postulated to be composed of four modular subunits (Sutcliffe 2008), which are ultimately linked together via phosphodiester bonds to form a multiantenna branching structure (Fig. 3B). Despite its structural complexity, it was hypothesized that a minimum of 11 glycosyltransferases would theoretically be required to compose all glycosidic linkages in the GBC, seven of which are encoded within the GBC gene cluster (Sutcliffe, Black and Harrington 2008). However, upon reexamination of the GBC structure (Michon *et al*. 1987, 1988, 1991), we have recognized the presence of two key structural elements, both beginning with GlcNAc: a stem oligosaccharide and a repeating unit (Fig. 3A, B). Following transfer of GlcNAc to the undecaprenylphosphate lipid carrier by the GbcO UDP-GlcNAc:lipid phosphate transferase, cytoplasmic synthesis of both of these subunits could be achieved by the glycosyltransferases encoded in the GBC gene cluster (unpublished observations). Identification of this repeat unit allows for a revised model of GBC assembly (see below) and solves the mystery of the two ‘missing’ GlcNAc transferases (Sutcliffe, Black and Harrington 2008).

In contrast to the invariable structure of the GAC, GBC, GCC and GGC in the respective streptococci, *L. lactis* strains can express structurally diverse CWP structures, correlating to diversity in the responsible gene cluster (Chapot-Chartier *et al*. 2010; Mahony *et al*. 2013; Ainsworth *et al*. 2014; Farenc *et al*. 2014). Rhamnose is not incorporated into the CWP of every strain (Ainsworth *et al*. 2014). Bioinformatics predict the presence of eight glycosyltransferases in the *L. lactis* pellicle gene cluster but for none of them a contribution or role has been experimentally addressed. Currently, the structure of the *E. faecalis* Epa awaits elucidation, making it difficult to speculate about the specific glycosyltransferases involved. However, genetic disruption of *epaB* and *epaN*, which encode putative glycosyltransferases (Fig. 5), changes immunoreactivity of Epa, indicating that these enzymes are involved in Epa biosynthesis (Xu, Murray and Weinstock 1998; Teng *et al*. 2002, 2009). Moreover, the Epa polysaccharide extracted from the Δ*epaB* mutant was incapable of incorporating rhamnose and instead included mannose, suggesting that *epaB* encodes a rhamnosyltransferase (Teng *et al*. 2009).

#### RhaCWP translocation and incorporation into the cell wall

Transport of big sugar complexes across cell membranes is energetically unfavorable and requires one of three mechanisms, i.e. synthase-dependent transporters, ABC transporters, or so-called ‘flippases’ (Cuthbertson, Kos and Whitfield 2010). The synthase-dependent pathway is least well defined and is involved in the formation of polysaccharides such as chitin, cellulose, hyaluronan and poly-*N*-GlcNAc. It was recently confirmed that a single protein, the synthase, executes both polymerization and export of the growing polysaccharide chain (Morgan, Strumillo and Zimmer 2013). ABC transporters translocate longer glycan chains and can be composed of a single protein (for example PglK; Perez *et al*. 2015) or of a two-protein complex consisting of a permease protein and an ATP-binding protein (Cuthbertson, Kos and Whitfield 2010). Finally, classical Wzx flippase systems generally transport oligosaccharide repeat units that are polymerized on the extracytoplasmic face of the membrane by a Wzy-like polymerase to complete the mature polysaccharide (Islam and Lam 2013). Interestingly, genes consistent with either the Wzx flippase and/or the ABC transport system seem to be present in all RhaCWP gene clusters (Fig. 5). Currently, the only experimental evidence supporting a role for the cognate ABC transport system is for *S. mutans* (Shibata *et al*. 2002). After heterologous expression of the first seven (*rmlD*-*rgpABCDEF*) genes in *E. coli* and subsequent disruption of the ABC transporter-encoding genes (r*gpCD*), the bacteria were unable to produce rhamnan (Shibata *et al*. 2002). This transport mechanism would resemble WTA translocation, which involves the TagG/H ABC transport system (Lazarevic and Karamata 1995).

The translocation of the GBC structure remains particularly enigmatic, given that assembly of a fully branched polymer before translocation would present a major challenge (Sutcliffe, Black and Harrington 2008). In contrast to the other RhaCWP gene clusters, the GBC gene cluster only contains a putative Wzx flippase gene (*gbs1482*) as well as two integral membrane proteins (encoded by *gbs1483* and *gbs1490*) that may act as accessory proteins (Sutcliffe, Black and Harrington 2008). Recognition that the GBC is composed of two structural elements (stem oligosaccharide and a repeat unit, Fig. 3B) instead of the previously predicted four elements, now allows for a revised route of synthesis. Flipping of these two building blocks (by GBS1482 and, presumptively, a second flippase) could allow assembly of the mature GBC on the extracytoplasmic face of the membrane. The integral membrane proteins (GBS1484, GBS1489 or GBS1490) can be proposed as the second flippase and/or assembly proteins. Assembly of repeating units requires formation of linkages between the GlcNAc and C3 of a terminal rhamnose in the growing chain, whilst the branch points are formed by repeating unit linkage to the C4 of the penultimate rhamnose. An attractive aspect of this revised proposed biosynthesis is that it removes the need for as yet unidentified cytoplasmic GlcNAc transferases. In addition, it would allow flipping of smaller units instead of the fully branched GBC, which is likely energetically more favorable. Thus, complete synthesis, translocation and assembly of the GBC can be predicted to occur through the action of GbcO and the proteins encoded in GBC gene cluster. Experimental evidence to support these hypotheses should now be sought.

Detailed biochemical analysis has demonstrated that the GAC and GBC are attached to the MurNAc moiety of peptidoglycan, similar to WTA in other Gram-positive bacteria (Swoboda *et al*. 2010). GAC is presumably connected to MurNAc through a phosphate containing bridge composed of one or more units of glycerol (Heymann, Manniello and Barkulis 1967), which would concur with the linkage of WTA to peptidoglycan in *S. aureus* (Swoboda *et al*. 2010). The transfer of nascent RhaCWP from the flipped lipid carrier onto the peptidoglycan is likely catalyzed by a member of the LytR-CpsA-Psr family. These proteins were initially identified to catalyze the attachment of WTA to peptidoglycan in *B. subtilis* (Kawai *et al*. 2011). This family of proteins is widespread throughout Gram-positive bacteria, with at least two family members present in the genomes of streptococci up to five in *E. faecalis* (Hubscher *et al*. 2008). The LytR-CpsA-Psr proteins appear to be highly redundant since lack of peptidoglycan-attached glycopolymers, such as capsule and WTA, only becomes apparent upon genetic mutation of all encoded enzymes (Kawai *et al*. 2011; Eberhardt *et al*. 2012; Chan *et al*. 2013, 2014). The LytR-CpsA-Psr phosphotransferases typically hydrolyze the phosphodiester linkage between the lipid-carrier and the first GlcNAc at the stem base of the polysaccharide (i.e. they hydrolyze the linkage created by the UDP-GlcNAc:lipid phosphate transferase such as GacO and GbcO) and attach the polymers to peptidoglycan via a phosphate ester linkage (Kawai *et al*. 2011; Eberhardt *et al*. 2012; Chan *et al*. 2013). Overall, it seems likely that LytR-CpsA-Psr proteins are involved in anchoring RhaCWP to the cell wall, but experimental proof is currently lacking.

#### Distribution of RhaCWP throughout bacteria: identification of additional RhaCWP gene clusters

As mentioned above, the Lancefield typing scheme is unable to discriminate bacteria up to the species level. The availability of genome sequences, as well as knowledge regarding RhaCWP gene clusters, provides an opportunity to gain insight into the distribution and potential structure of RhaCWP among Gram-positive bacteria. For example, *S. castoreus* was noted to react with Group A-specific antisera and indeed its draft genome contains a biosynthetic locus syntenous with that of the GAC of *S. pyogenes* (Table S1, Supporting Information). However, the presence of an additional glycosyltransferase compared to the GAC gene cluster in *S. pyogenes* suggests some fine structural variation. Likewise, the GBC has been reported to be expressed by different streptococcal species most notably *Streptococcus porcinus*, *Streptococcus pseudoporcinus*, *Streptococcus troglodytidis* and *Streptococcus plurextorum*. Correspondingly, the genomes of *S. pseudoporcinus* and *S. porcinus* contain a fully syntenous GBC biosynthetic gene cluster except for the lack of a *gbs1485* orthologue (unpublished observations). Presumably, the expressed structures only lack one of the monosaccharide rhamnose side-branches present in the GBC repeating unit (Fig. 3B) (Sutcliffe, Black and Harrington 2008), but can still make the linear trirhamnosyl immunodominant epitope with a terminal rhamnose that is detected by Group B-specific serotyping (Curtis and Krause 1964b). In contrast, *Streptococcus thoraltensis* contains a GBC-variant gene cluster that lacks orthologues of two of the predicted rhamnosyltransferases, *gbs1481* and *gbs1485*, present in *S. agalactiae* (Table S2, Supporting Information) and this species is non-groupable by Lancefield serotyping assays. Instead of *gbs1481* and *gbs1485*, the *S. thoraltensis* locus harbors two additional glycosyltransferases absent in the otherwise syntenous locus of *S. agalactiae* (Table S2, Supporting Information)*.* Absence of a *gbs1481* orthologue is likely responsible for abrogated cross-reactivity in the Group B antigen serotyping tests due to the loss of the terminal rhamnose from the dominant trirhamnosyl epitope. This analysis therefore suggests that *S. thoraltensis* is capable of synthesizing a RhaCWP that is a structural variant of the GBC. Increased availability of genome sequences for streptococci and related species will help identify additional *rmlD*-linked gene clusters for RhaCWP biosynthesis that are consistent with either known Lancefield serotyping reactions (as exemplified here by *S. castoreus*) or from which variant or novel RhaCWP structures can be predicted.

### Physiological role of RhaCWP

Since their identification and structural characterization from the 1930s onwards, the biological roles of the Lancefield Group antigens or other RhaCWP have received little attention. It was long thought that Group-specific antigens were only of structural importance (McCarty 1952). Indeed, complete loss of RhaCWP expression through genetic mutation results in severe growth and cell division abnormalities (Fig. 1) and can be essential under competing conditions (McCarty 1952; Tsukioka *et al*. 1997a; Caliot *et al*. 2012; van der Beek *et al*. 2015). Caliot *et al*. (2012) targeted the UDP-GlcNAc:lipid phosphate transferase *gbcO* (*gbs0136*) of *S. agalactiae* to initiate functional studies on the GBC. The resulting GBC-negative *S. agalactiae* strain was devoid of cell wall rhamnose and phosphate and lost expression of the pellicle structure (Caliot *et al*. 2012) (Fig. 2). The loss of GBC was associated with major morphological and cell growth defects, including mislocated septa and defects in cell division and separation, which resulted in the formation of very long chains (Caliot *et al*. 2012). This phenotype corresponds to previous descriptions of a stable opacity variant of *S. agalactiae* that had lost GBC expression and displayed growth and morphological defects (Pincus *et al*. 1992, 1993). For this spontaneous opaque *S. agalactiae* mutant strain the underlying genetic defect has never been clarified. For the *gbcO* mutant strain, the defects result from reduced levels of highly cross-linked peptidoglycan and mislocalization of the important peptidoglycan hydrolase PcsB (Caliot *et al*. 2012), a protein required for streptococci cell wall separation (Reinscheid *et al*. 2003; Sham *et al*. 2011). The latter observation is reminiscent of studies in *S. aureus*, where the preferential localization of the major autolysin Atl is lost in absence of WTA (Schlag *et al*. 2010). Collectively, these observations support a role of GBC in cell wall homeostasis of *S. agalactiae*. In line with observations in the *S. agalactiae gbcO* mutant (Caliot *et al*. 2012), pharmacological inhibition of the *S. pyogenes* enzyme GacO (encoded by *M5005_Spy0240*) by tunicamycin resulted in depletion of GAC from the cell wall, increased mutanolysin susceptibility and increased chain length as a result of cell separation defects (van Sorge *et al*. 2014). However, interpretations from these studies may be obscured by the interconnection between biosynthesis of Group-specific antigens and that of other cell wall glycopolymers, including peptidoglycan, since most glycoconjugates use undecaprenylphosphate as a carrier for biosynthesis. Coordinated regulation between biosynthesis of different glycoconjugates is further supported by the observation that the *S. agalactiae gbcO* mutant increases capsule production suggesting a coordinated regulation between the two glycoconjugates (Beaussart *et al*. 2014). Knowledge regarding the interrelatedness of glycoconjugate biosynthesis pathways is relevant for the design of new antibiotics since interference of such connected pathways may have synergistic effects as recently demonstrated for WTA and peptidoglycan biosynthesis (Sewell and Brown 2014).

### RhaCWP as phage receptors

In addition to their significant role in cell wall architecture, it is appreciated that RhaCWP are important phage receptors for many species. This again highlights a parallel with WTA in other Gram-positive bacteria where WTA is critical to phage-mediated horizontal gene transfer (Baptista, Santos and Sao-Jose 2008; Brown *et al*. 2012; Winstel *et al*. 2014). For several streptococci, the specificity of phage adsorption correlates to the side chain anchored to the rhamnan backbone. Indeed, the (GalNAc)_2_ side chain of the GCC serves as an attachment site for Group C1 bacteriophage in Group C *Streptococcus* (Krause 1957; Fischetti and Zabriskie 1968). Correspondingly, a Group C-variant strain, which completely lacks the immunodominant (GalNAc)_2_ epitope, was isolated from Group C *Streptococcus* that survived exposure to Group C1 lytic phages (Krause 1963). Also for *S. pyogenes*, the GAC-specific GlcNAc terminal moiety appears to be involved in both lytic and temperate phage adsorption although additional unidentified cell wall factors are also involved (Fischetti and Zabriskie 1968). Finally, specific phages recognize the α-1,2-linked glucose side chain of serotype c *S. mutans* strains (Shibata, Yamashita and van der Ploeg 2009). For *L. lactis*, selection of phage resistant strains from a random insertional mutagenesis library originally identified the presence and genetic locus of the RhaCWP (Dupont *et al*. 2004). Moreover, the precise structure of the RhaCWP dictates bacteriophage sensitivity (Dupont *et al*. 2004; Mahony *et al*. 2013; Ainsworth *et al*. 2014). Finally, structural changes in *E. faecalis* Epa through genetic manipulation greatly affect phage sensitivity despite similar adsorption levels (Teng *et al*. 2009). Phage dynamics within the bacterial population will impact fitness and may also be important for horizontal gene transfer, even across long phylogenetic distances (Winstel *et al*. 2013), affecting virulence or antibiotic resistance of pathogens. Thus the interactions of phages with RhaCWP are likely to impact on bacterial population structure. In the case of *L. lactis*, knowledge regarding the molecular mechanisms of phage adsorption and infection may benefit the food industry.

### Role of RhaCWP in virulence

The localization of RhaCWP at the host–pathogen interface suggests that their biological function might be broader than a role in cell wall biogenesis. Similarly for WTA, evidence is accumulating for its role in virulence by increasing adherence and immune evasion (Carvalho *et al*. 2015; Winstel *et al*. 2015). Recent studies in *S. pyogenes* and *E. faecalis* now highlight that subtle modifications to the RhaCWP structure, which do not impact bacteria growth, can significantly impact virulence (Xu *et al*. 2000; Teng *et al*. 2009; van Sorge *et al*. 2014). For *E. faecalis*, disruption of *epaB*, *epaE*, *epaM* and *epaN*, which may completely eliminate Epa expression or only modify its structure, all caused significant attenuation in a mouse peritonitis model (Xu *et al*. 2000; Teng *et al*. 2009). Reduced virulence correlated with increased phagocytic uptake and clearance by neutrophils (Teng *et al*. 2002). Similarly in *L. lactis*, loss of the pellicle results in 10-fold more efficient uptake by macrophage cell lines compared to wild-type bacteria (Chapot-Chartier *et al*. 2010). These results therefore indicate that the pellicle can exert an anti-phagocytic effect both for *L. lactis* and *E. faecalis*. For *S. pyogenes*, structure–function studies focused on the role of the GAC GlcNAc side chain, which was selectively removed through genetic mutation of the glycosyltransferase-encoding gene *gacI* (van Sorge *et al*. 2014)*.* The *gacI* mutant bacteria still expressed the rhamnan backbone but did not display apparent cell wall abnormalities. However, bacteria were increasingly susceptible to innate immune clearance by neutrophils and antimicrobial components (van Sorge *et al*. 2014). Moreover, virulence of this genetically engineered Group A-variant strain was significantly attenuated in two animal models (van Sorge *et al*. 2014). Again, this indicates that specific epitopes of RhaCWP increase bacterial immune resistance, although the mechanism has not been well defined. In addition to increased immune resistance, RhaCWP may modulate host immune responses by targeting specific lectin receptors (carbohydrate-recognizing pattern-recognition receptors) (Sancho and Reis e Sousa 2012). Human lectins regulate fundamental immunological processes but also directly engage microbial carbohydrates, linking pathogen recognition to appropriate immune responses. Although this often promotes bacterial clearance, lectin targeting can also promote bacterial survival by skewing immune reponses (van Vliet *et al*. 2009). Research in this area should provide insight whether the virulence-promoting effect of RhaCWP occurs through interaction with lectin receptors. Alternatively, decoration of the rhamnan backbone or rhamnose moieties by common sugars such as GlcNAc, glucose and GalNAc may be a strategy for microorganisms to avoid immune recognition. This is relevant since the absence of rhamnose in humans makes rhamnose an attractive pattern-associated molecular pattern (PAMP). Indeed, in fish and invertebrates, rhamnose is targeted by the innate immune system through germ-line encoded pattern-recognition receptors called rhamnose-binding lectins (Ogawa *et al*. 2011; Ng *et al*. 2014). Interestingly, rhamnose-binding lectins agglutinate both Gram-positive and Gram-negative bacteria through interaction with glycan structures such as LPS or lipoteichoic acid in the bacterial cell wall (Tateno *et al*. 2002; Cammarata *et al*. 2014; Ng *et al*. 2014). Rhamnose-binding lectins are also involved in inflammatory responses through the induction of cytokines (Watanabe *et al*. 2009). Humans lectins with specificity for rhamnose have not been identified yet, but their existence might be anticipated given the estimated presence of over 150 glycan-binding proteins in humans, many with uncharacterized ligand specificity (Zelensky and Gready 2005; Drickamer 2014). Overall, the role of RhaCWP in pathogenesis and cell physiology is just starting to be appreciated. Further investigations are likely to unravel new bacterial immune evasion strategies but may also contribute to new insights into innate immune responses and recognition.

### Rhamnose containing capsules in Gram-positive bacteria

Rhamnose is not just incorporated in RhaCWP, but is also present in capsular polysaccharides. We consider this structure distinct from RhaCWP given their localization in the cell wall; RhaCWP are interpolated within the peptidoglycan wall layer, whereas capsular polysaccharides are typically the outermost layers of the cell envelope. Nevertheless, it is worth noting that several clinically relevant Gram-positive bacteria synthesize rhamnose-containing capsules. The significance of the *S. pneumoniae* capsule as a key virulence factor has been established since the 1928 landmark ‘Griffiths experiment’ and the capsular polysaccharides are major protective antigens utilized in current vaccine formulations (Geno *et al*. 2015). Of the 41 (out of 46) serogroups of pneumococcal capsule for which carbohydrate composition or structural information is available, 17 of these (∼40%) contain rhamnose, including those of clinically significant serogroups such as 6, 19 and 23. The capsular biosynthetic loci for these serogroups all contain *rmlABCD* genes (Bentley *et al*. 2006). The presence of these genes in the biosynthetic loci for which capsular polysaccharide structures are not yet available or may be incomplete (e.g. serogroups 21, 40 and 48) suggests additional structures also contain rhamnose. Thus, rhamnose biology is likely of significance in much of the *S. pneumoniae* population and at least nine of the serotype antigens included in the current 23-valent vaccin are rhamnose-containing polysaccharides (Geno *et al*. 2015).

In addition to *S. pneumoniae*, *S. agalactiae* strains belonging to serotype VIII contain rhamnose within the polysaccharide repeating unit (Kogan *et al*. 1996; Cieslewicz *et al*. 2005). This serotype remains relatively rare globally but has been reported to be of significance in some population groups, notably in Japan and the Pacific (Lachenauer *et al*. 1999; Edmond *et al*. 2012).

### Therapeutic and technological applications

Increased knowledge regarding the biosynthesis and function of RhaCWP could aid the development of new antimicrobial agents but may also have applications in metabolic engineering to optimize food production (Chapot-Chartier 2014) or glycoconjugate production for medical purposes (Jaffe *et al*. 2014). Clearly, the L-rhamnose biosynthesis pathway holds promise for antimicrobial drug targeting, given the loss of virulence and/or viability upon rhamnose depletion in a wide range of Gram-positive and Gram-negative bacteria. More importantly, lack of L-rhamnose in humans should preclude off-target effects lowering risks of unwanted side effects. Thus far, several inhibitors screens for Rml enzymes have been initiated, resulting in only one RmlA inhibitor targeting *P. aeruginosa* with some marginal activity against *M. tuberculosis* (Alphey *et al*. 2013). In addition to targeting rhamnose, inhibition of other steps in the RhaCWP biosynthesis pathway could also be of interest. Although this may not immediately kill the bacterium, it may act as anti-virulence agent, increasing susceptibility to host defense mechanisms such as phagocytic clearance (Nizet 2015). A similar strategy is currently exploited for *S. aureus*, where inhibition of the UDP-GlcNAc:lipid phosphate transferase TarO is not detrimental to the bacterium, but render the bacterium avirulent (Sewell and Brown 2014). An additional effect of TarO inhibition is the re-sensitization of resistant bacteria to β-lactam antibiotics due to an interaction between the WTA and peptidoglycan biosynthesis pathways in *S. aureus* (Campbell *et al*. 2011). Similar synergy may occur between conventional antibiotics and inhibitors of the RhaCWP pathway.

RhaCWP are also attractive vaccine candidates due to their conserved and constant expression in species of medical importance, such as *S. pyogenes* and *S. agalactiae*. Indeed, different strategies are currently explored to develop protective vaccines against these streptococcal species (Dale *et al*. 2013; Steer, Dale and Carapetis 2013; Nuccitelli, Rinaudo and Maione 2015). For both pathogens, much research has focused on type-specific vaccine strategies, i.e. a capsule-conjugate vaccine for *S. agalactiae* (Johri *et al*. 2006; Nuccitelli, Rinaudo and Maione 2015) and a multivalent M-protein vaccine for *S. pyogenes* (Dale *et al*. 2013; Steer, Dale and Carapetis 2013). A more elegant and globally effective approach would employ a vaccine antigen that is universally expressed on all strains in an invariant manner. Indeed, the GBC is immunogenic in rabbits but antibodies raised are not protective in a newborn mouse model (Marques *et al*. 1994), likely due to shielding of the GBC by the polysaccharide capsule. More encouraging are the results with regard to the GAC as a universal *S. pyogenes* vaccine antigen. Conjugate vaccines of either isolated or synthetic GAC protect mice from subsequent infection after active and passive immunization (Sabharwal *et al*. 2006; Kabanova *et al*. 2010). However, there is controversy with regards to safety in the use of the native GAC for vaccine purposes, since several groups have indicated a role for anti-GlcNAc antibodies in the pathogenesis of rheumatic fever (Goldstein *et al*. 1968; Ayoub and Dudding 1970; Kirvan *et al*. 2003). Recent elucidation of the molecular pathway for GlcNAc side chain formation allows for the development of an alternate vaccine antigen consisting of the polyrhamnose backbone of the GAC (van Sorge *et al*. 2014). Anti-rhamnan antibodies raised against the GlcNAc-deficient GAC enhanced phagocytic killing of multiple M-serotypes *in vitro* and protected mice from lethal challenge with wild-type *S. pyogenes* through passive immunization (van Sorge *et al*. 2014). Of interest is the observation that some streptococci also release RhaCWP into their surroundings. Although it is unclear whether this involves an active mechanism of release, it possibly has implications for the efficiency of vaccine strategies. Overall, further exploration on the application of RhaCWP for vaccination purposes is warranted.

Finally, dissection of the RhaCWP biosynthesis pathway could benefit food production, most notably the dairy industry that uses lactic acid bacteria for food fermentations (Chapot-Chartier 2014). Phage infection of these fermenting cultures results in product variations but can also lead to huge economic losses (Samson and Moineau 2013). The selection or engineering of phage-resistant strains is therefore of considerable interest. The role of RhaCWP in phage–host interaction has opened up new possibilities for the development of bacteriophage insensitive mutants for food production purposes. In addition to food preparation, polysaccharides often have cosmetic, pharmaceutical and biomedical applications. Elucidation of specific transferase activities could be used towards metabolic engineering of new materials or compounds with interesting biological or physical properties. In particular, the incorporation of rare sugars such as rhamnose and uronic acids is a rather unexplored area but may be of interest to different areas including the biomedical field (Roca *et al*. 2015).

## CONCLUDING REMARKS

Despite their long history in streptococcal diagnostics, investigations on the biological roles and possible applications of RhaCWP have lagged behind. Genome sequencing has initiated genetic studies to elucidate structure–function relationships of RhaCWP, highlighting their critical importance in proper cell wall architecture and pathogenesis. Their indispensable nature identifies the RhaCWP biosynthesis pathway as an attractive therapeutic target for antimicrobial drug development. Spin offs will likely find applications in the area of metabolic engineering for food production and biomedical purposes.

## Supplementary Material

Supplementary Data
