# Supplementary material for: Bacterial glycobiology: rhamnose-containing cell wall polysaccharides in Gram-positive bacteria
Source: FEMS Microbiol Rev. 2016 Mar 13;40(4):464–79. doi: 10.1093/femsre/fuw006 (PMC4931226; doi:10.1093/femsre/fuw006)
Supplement: Supplementary Data [file fuw006_supplementary_data.zip › Supplementary_Tables.doc]

**Supplementary Table 1. Gene locus for biosynthesis of a Group A carbohydrate variant in the genome of *Streptococcus castoreus.***

| ***S. castoreus*^1^** | **Function (with Pfam family matches)** | **% Identity** | **% Cover** | ***S. pyogenes* M1** |
| --- | --- | --- | --- | --- |
| VBIStrCas272379_0442 | PF01546 peptidase | 84 | 99 | Spy_0799 |
| VBIStrCas272379_0443 | PF09913 (DUF2142) membrane protein (flippase and/or assembly) | 70 | 100 | Spy_0798 |
| VBIStrCas272379_0444 | PF01943 Polysaccharide biosynthesis protein (flippase) | 75 | 99 | Spy_0797 |
| VBIStrCas272379_0445 | PF10066 (DUF2304) membrane protein (flippase and/or assembly) | 76 | 100 | Spy_0796**^2^** |
| VBIStrCas272379_0446 | PF00535 Glycosyltransferase family 2 | 84 | 100 | Spy_0794**^2^** |
| VBIStrCas272379_0447 | PF00535 Glycosyltransferase family 2 | - | - | No hit**^3^** |
| VBIStrCas272379_0448 | PF00884 sulfatase/LtaS phosphoglycerol transferase | 70 | 99 | Spy_0793 |
| VBIStrCas272379_0449 | PF05045 Rhamnan synthesis protein F | 75 | 100 | Spy_0792 |
| VBIStrCas272379_0450 | PF00535 Glycosyltransferase family 2 | 70 | 100 | Spy_0791 |
| VBIStrCas272379_0451 | PF00005 ABC transporter; ATPase polysaccharide/ polyol phosphate transport system | 86 | 100 | Spy_0790 |
| VBIStrCas272379_0452 | PF01061 ABC-2 type transporter permease component | 90 | 94 | Spy_0789 |
| VBIStrCas272379_0453 | PF00535 Glycosyltransferase family 2 | 84 | 99 | Spy_0787 |
| VBIStrCas272379_0454 | PF09314 (DUF1972) Glycosyltransferase (putative ß-rhamnosyltransferase) | 82 | 100 | Spy_0786**^4^** |
| VBIStrCas272379_0455 | PF04321 RmlD; dTDP-4-dehydrorhamnose reductase | 88 | 100 | Spy_0784**^4^** |
| VBIStrCas272379_0456 | PF01883 DUF59, conserved hypothetical protein | 84 | 100 | _0783 |

**^1^** Annotation according to PATRIC genome annotation service, searchable at <https://www.patricbrc.org/portal/portal/patric/Home>

**^2^** There is no Spy_0795 annotated between these two genes

**^3^** 76% identical to a putative *Streptococcus ictaluri* GTF

**^4^** There is no Spy_0785 annotated between these two genes

**Supplementary Table 2. Gene locus for biosynthesis of a Group B carbohydrate variant in the genome of *Streptococcus thoraltensis.***

| ***S. thoraltensis*** | **Function (with Pfam family matches)** | **% Identity** | **% Cover** | ***S. agalactiae* NEM316** |
| --- | --- | --- | --- | --- |
| WP_018379936.1 | Glycosyltransferase family 1, putative α-galactosyltransferase | 58 | 97 | GBS1480 |
|  | No orthologue (missing putative rhamnosyltransferase) | - | - | GBS1481 |
| WP_018379937.1 | PF01943 Polysaccharide biosynthesis protein (flippase) | 68 | 99 | GBS1482 |
| WP_018379938.1 | PF09913 (DUF2142) membrane protein (flippase and/or assembly) | 54 | 100 | GBS1483 |
| WP_018379939.1 | PF00535 Glycosyltransferase family 2 | 63 | 99 | GBS1484 |
|  | No orthologue (missing putative rhamnosyltransferase) |  |  | GBS1485 |
| WP_018379940.1 | PF01370 dTDP-glucose 4,6-dehydratase | 64 | 100 | GBS1486 |
| WP_018379941.1 | PF01128 2-C-methyl-D-erythritol 4-phosphate cytidylyltransferase | 78 | 100 | GBS1487 |
| WP_018379942.1 | PF04991 LicD family protein | 65 | 99 | GBS1488 |
| WP_018379943.1 | Conserved hypothetical integral membrane protein | 36 | 99 | GBS1489 |
| WP_018379944.1 | PF10066 (DUF2304) membrane protein (flippase and/or assembly) | 71 | 97 | GBS1490 |
| WP_018379945.1 | PF00535 Glycosyltransferase family 2 | 69 | 100 | GBS1491 |
| WP_018379946.1 | PF00535 Glycosyltransferase family 2 | 65 | 99 | GBS1492 |
| WP_018379947.1 | PF00535 Glycosyltransferase family 2 | 49 | 91 | GBS1492 |
| WP_018379948.1 | PF09314 (DUF1972) Glycosyltransferase (putative ß-rhamnosyltransferase) | 64 | 100 | GBS1493 |
| WP_018379949.1 | PF04321 RmlD; dTDP-4-dehydrorhamnose reductase | 86 | 98 | GBS1494 |
| WP_018379950.1 | PF00535 Glycosyltransferase family 2 | 68 | 100 | GBS1545 |
| WP_018379951.1 | PF01883 DUF59, conserved hypothetical protein | 75 | 100 | GBS1495 |

**^1^** Genbank accession number
